# Objectively measured adherence to physical activity among patients with coronary artery disease: Comparison of the 2010 and 2020 World Health Organization guidelines and daily steps

**DOI:** 10.3389/fcvm.2022.951042

**Published:** 2022-09-28

**Authors:** Prisca Eser, Nathalia Gonzalez-Jaramillo, Selina Weber, Jan Fritsche, Riccardo Femiano, Charlotte Werner, Flurina Casanova, Arjola Bano, Oscar H. Franco, Matthias Wilhelm

**Affiliations:** ^1^Department of Cardiology, Inselspital, Bern University Hospital, University of Bern, Bern, Switzerland; ^2^Institute of Social and Preventive Medicine, University of Bern, Bern, Switzerland; ^3^Graduate School for Health Sciences, University of Bern, Bern, Switzerland; ^4^Department of Health Sciences and Technology, ETH Zurich, Zürich, Switzerland

**Keywords:** physical activity, guidelines, accelerometer, percutaneous coronary intervention, step counting

## Abstract

**Background:**

Tailored recommendations for patients after percutaneous coronary interventions (PCI) need physical activity (PA) to be objectively measured and assessed for adherence to guidelines. The recent WHO guidelines removed the daily recommended bout duration, while the potential impact of this change on patients after PCI remains unclear.

**Aim:**

We evaluated prevalence estimates of adherence to PA recommendations among patients after PCI across the 2010 [≥30 min moderate- to vigorous-intensity PA (MVPA) at ≥ 10-min bout duration] and 2020 WHO guidelines (≥30 min of MVPA of any bout duration), as well as 7,500 and 10,000 steps.

**Methods:**

We conducted an observational longitudinal single-center study with patients after PCI for chronic or acute coronary syndrome (ACS); maximal age 80 years. Wrist-worn accelerometers recorded participants’ PA data from the evening of hospital discharge over the next 18 days.

**Results:**

We analyzed data from 282 participants with sufficient minimum wear time (7 days of ≥12 h), including 45 (16%) women; and 249 (88%) with ACS. Median wear time was 18 (17, 18) days. Median participant age was 62 (55, 69) years. Fifty-two participants (18.4%) fulfilled 2010 WHO guidelines and 226 (80.1%) fulfilled the 2020 WHO guidelines. Further, 209 (74.1%) participants achieved ≥7,500 steps/day and 155 (55.0%) performed ≥10,000 steps/day.

**Conclusion:**

Among participants after PCI, most MVPA was accumulated in bouts <10 min, leading to a fourfold discrepancy between participants fulfilling the 2010 and 2020 WHO PA recommendations. The number of steps/day may be a valid proxy to recent WHO PA recommendations as it is not dependent on the bout-length definition.

**Clinical trial registration:**

[ClinicalTrials.gov], identifier [NCT04663373].

## Introduction

Cardiovascular disease (CVD) remains the leading cause of death globally (1). Recent studies have found that lower levels of objectively measured physical activity (PA) were associated with higher rates of hospital readmission and adverse outcomes among patients after acute myocardial infarction, cardiac surgery, or decompensated heart failure (2–4). Similarly, daily steps have been associated with CVD risk factors and cardiometabolic outcomes (5, 6). In addition, a curvilinear relationship between PA volume and health benefits has been demonstrated, suggesting that the most significant reduction in morbidity and premature death were achieved with increases in PA among patients with coronary heart disease (CHD) (7) and healthy people at the lowest level in the spectrum of PA (8). A recent meta-analysis on PA trajectories among patients with CHD provided evidence supporting the benefits of maintaining or adopting an active lifestyle to improve survival and the possible harms of decreasing PA (9). For instance, compared to always-inactive patients, the pooled risk of all-cause mortality was 50% lower in those who remained active [HR (95% CI) = 0.50 (0.39–0.63)], 45% lower in those who were inactive but became active [0.55 (0.44–0.7)], and 20% lower in those who were active but became inactive [0.80 (0.64–0.99)] (9). PA is a foundational therapy for patients with CHD. Therefore, it is crucial to identify patients with low levels of PA, increase their PA, and facilitate a tailored cardiac care approach (10).

According to WHO’s 2020 “Guidelines on Physical Activity and Sedentary Behaviour,” adults should be physically active for 150–300 min per week with moderate intensity, 75–150 min per week with vigorous intensity, or an equivalent combination of the two to achieve substantial health benefits (11, 12). Moderate- to vigorous-intensity PA (MVPA) has been defined as a metabolic demand of greater than three times resting (3 METs) (12). PA is most commonly assessed by commercial accelerometers calibrated against measurements by metabolic carts so accelerations during activities with >3 METs are classified as MVPA (13, 14). Some calibration studies of accelerometers used steady-state activities, such as walking, running, and cycling, which require >3 METs when performed continuously for longer than 1–2 min when metabolism has reached a steady state (13, 14). When these accelerations occur for only a few seconds, they do not lead to energy consumption >3 METs. Therefore, WHO 2010 guidelines recommended performing MVPA in bouts of 10 min when the threshold of MVPA had to be reached 80% of the time. However, this bout requirement was lowered in the WHO 2020 guidelines because new evidence suggested that MVPA bouts <10 min also have beneficial effects on health and were associated with reduced all-cause mortality (15). The consequence of not requiring a minimal bout duration is that accelerations of single movements may be counted toward MVPA or a step count goal even if a person never exceeds 3 METs during an entire day. Therefore, the same volume and intensity of activities may result in varying minutes with MVPA when measured and analyzed by different commercial accelerometers whose algorithms are not available to the user.

Since walking is often the chosen exercise for people with heart disease, an alternative criterion to quantify PA is the number of steps; (6) steps per day is a practical PA measure because it is an easy-to-understand recommendation (16, 17). The commonly used artificial recommendation of 10,000 steps per day—promoted by a Japanese pedometer company in the 1960s (18)—was not based on scientific evidence, yet it has been used as the threshold value for providing health benefits in several studies (6, 19–22). Although achieving 10,000 steps/day was associated with meeting PA guidelines, (20) there is no conclusive evidence about how many steps per day are required for better health outcomes (16). For instance, Lee et al. found that hazard ratios associated with mortality continuously decreased with an increasing mean of daily steps among older women, leveling off at around 7,500 steps/day (16). Other studies supported a threshold of 7,500 steps per day for patients with cardiac conditions to reduce CVD risk factors, CVD morbidity, and mortality, as well as all-cause mortality (5, 6, 23).

For physically inactive patients with CVD, the usage of activity trackers has been recommended by the newest ESC guidelines for patients with CVD. However, using different evidence-based PA criteria may influence prevalence, therapy recommendations, and tools to promote PA among these patients. Therefore, comparing prevalence across guidelines may help determine actionable recommendations for patient benefit. Thus, we evaluated prevalence estimates of adherence to PA recommendations across different guidelines among participants with coronary artery disease who recently underwent percutaneous coronary interventions (PCI) and wore a wrist accelerometer over 18 days after hospital discharge.

## Materials and methods

### Study population

Our study is a substudy of the Prognostic Impact of Physical Activity Patterns After Percutaneous Coronary Intervention (PIPAP) study (ClinicalTrials.gov identifier: NCT04663373)—a prospective observational single-center study that monitors patients’ PA and assesses the potential of acceleration and steps parameters for risk quantification. The PIPAP study was approved by the Ethics committee of the Canton of Bern, Switzerland.

We recruited consecutive patients hospitalized for PCI after acute or chronic coronary syndrome (ACS, CCS) on their day of discharge or one day before discharge from December 2020 to March 2022. Substudy participants were provided with a wrist-worn accelerometer; a study information sheet, including an informed consent form; and a pre-addressed, prepaid envelope to return the signed consent form and accelerometer after the study period. Participants were asked to wear the accelerometer for 18 successive days starting from the evening of the day of their discharge from the hospital. We included patients who were aged <80 years and eligible for ambulatory cardiac rehabilitation, which *de facto* excluded patients who are frail or cognitively impaired. We also excluded study participants who did not record PA data for ≥7 days for ≥12 h.

### Physical activity monitoring

Participants wore tri-axial accelerometers (Axivity AX-3, Axivity Ltd., Newcastle, UK) on their non-dominant wrist for 18 days. We programmed the devices using AX3 GUI V43 (24)—an open-source software—to record tri-axial accelerations of ±8 g at 50 Hz for 18 days starting on the evening of the day of the participant’s hospital discharge. We chose 18 days to capture at least 14 days of PA data from participants who were transferred to another hospital before returning home. Transfer to another hospital usually delayed hospital discharge by 1–3 days.

### Physical activity data processing

Using AX3 GUI V43, we downloaded PA data as continuous wave accelerometer (.cwa) files and then processed the PA data with the research-driven open-source R package GGIR (version 2.4.0) (25, 26). We derived participants’ demographic (age and sex) and PCI data from the participating clinic’s patient information system.

We calculated the movement component from the raw acceleration data using the default acceleration metric of the package—the Euclidean norm (vector magnitude) minus one (ENMO). It describes the raw tri-axial acceleration data conversion into an omnidirectional measure of body acceleration (27). The resulting ENMO values were expressed in gravity-based acceleration units [milligravity units (mg)] averaged over 5 s epochs.

We defined the following activity domains: <25 mg for inactivity; 25–99 mg for light PA; and ≥100 mg for MVPA, according to O’Donnell et al. (28). Sleep was also identified by the GGIR algorithm as documented and validated by van Hees et al. (29). Time spent in different PA domains was accrued in 1-min bouts. During analysis, we conducted autocalibration using local gravity as the reference, and we determined non-wear time over a window size of 60 min with a 15-min sliding window (30, 31).

While we derived activity parameters directly from GGIR, we determined steps by a Windowed Peak Detection open-source algorithm (Verisense_step_algorithm, last updated: 14.04.2021) based on Gu et al.’s (32) design and implemented for use in combination with the GGIR R package available on GitHub (33). We used validated input parameters for the step algorithm from a previous study of 22 participants during an outdoor physiotherapy session from the PIPAP study population (34).

### Calculating parameters

We derived the following activity parameters from the GGIR package. First, the algorithm was set to calculate data from midnight to midnight. Next, we calculated the daily minutes with MVPA, inactivity, and sleep time. Further, we computed mean acceleration values in mg over each 24-h cycle. As Rowlands et al. recently suggested, (35) we determined minimal accelerations during the most active 2, 30, and 60 min in mg to compare with studies using different activity thresholds. We also calculated minutes in MVPA as bouts of at least 10 min with 80% of the 5 s epochs having accelerations over the MVPA threshold.

The step counting algorithm Verisense returned the number of daily steps for each valid day (i.e., wear time ≥12 h). Additionally, we calculated cadences for each minute from the meta-data Verisense derived, which included the number of steps for each 5 s epoch. We calculated the mean cadence over the whole 24-h cycle from these values. Moreover, we calculated daily minutes with ≥100 steps/min and 0 steps/min (5). We also determined mean cadences for the most active 1, 30, and 60 min, as proposed by Tudor-Locke et al. (36). We summarized all parameters as the mean of each participant’s overall valid days and the median of all participants.

### Statistical analysis

We performed all analyses with R Studio (Version 1.4.1106-5). We calculated descriptive statistics reporting the number of participants and percentages of all participants and medians with first and third quartile for continuous activity parameters due to their primarily non-parametric distribution. We performed linear regressions for MVPA based on 1-min bouts and MVPA based on 10-min bouts with daily steps using the lm function. We calculated the proportions of adherence to the 2010 and 2020 WHO guidelines and daily steps for the total sample and for subgroups according to sex, median age of the sample (<62 versus ≥62 years old), and clinical presentation of the disease (ACS versus CCS).

## Results

### Study participants

**FIGURE 1:**
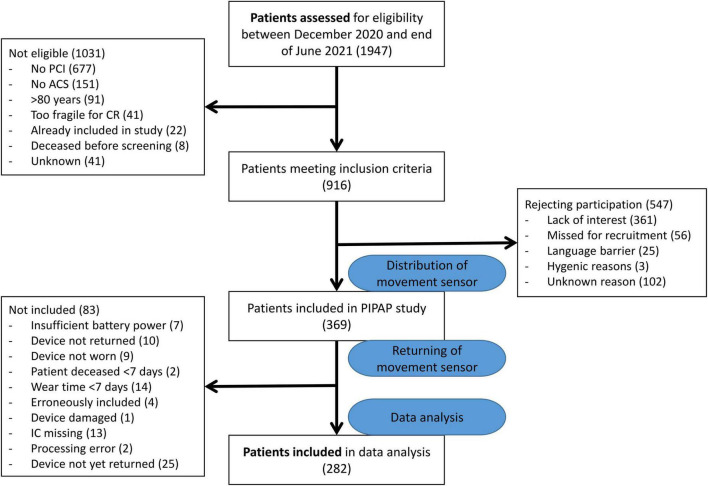
Patient flow.

Of the 916 patients who met inclusion criteria within our 16-month recruitment period, 369 patients (40.3%) agreed to participate in the study (Figure 1). We excluded 87 of those 369 participants. During the observational period, two participants (0.5%) died before completing 7 days of wear-time; ten participants (2.7%) never returned the accelerometer, and nine participants (2.4%) never wore the accelerometers. Twenty-seven additional participants met exclusion criteria: 10 participants had <7 days of ≥12-h wear time, four participants were aged >80 years, and 13 participants (3.5%) did not send the informed consent forms. Seven (1.9%) participants’ accelerometers had insufficient battery power. Devices of 25 participants had not yet been sent back and received by us. This resulted in 44 patients (11.9%) who were non-compliant with the study protocol. Consequently, we performed our data analysis with 282 valid recordings (76.4%).

Of the 282 participants with valid recordings, the median age was 61.5 (first quartile 55, third quartile 69) and 46 (16.1%) were women (Table 1). Thirty-three participants had CCS and 249 participants had ACS (88.3%). A third of all participants started recording on day 1 after PCI (PCI was on day 0), the majority started recording on the second day (75.9%) and by day 3 88.7% had started their recording. Therefore we included all recorded days as of day 2. The median number of days of device wear time ≥12 h was 18 (17, 18), and most participants (79.4%) still recorded day 18, while only 48.9% of patients still recorded day 19 after PCI. Between day 3 and day 18, data was available for at least 87.9% of all patients.

**TABLE 1:** Activity parameters of groups according to different gradients in MVPA and mean time spent in MVPA.

Group	All patients		2020 WHO guidelines (MVPA ≥30 min)		2010 WHO guidelines (MVPA ≥30 min, bouts ≥10 min)		Steps ≥7,500		Steps ≥10,000	
Number of patients	282		226		52		209		155	
Number of female patients (%)	45 (16.1)		34 (15.0)		4 (7.7)		33 (15.8)		27 (17.4)	
Age (years)	61.5	(55, 69)	61	(54, 68)	60	(55, 67)	61	(55, 68)	61	(56, 69)
LPA time (min/day)	242	(193, 293)	255	(213, 303)	259	(223, 296)	260	(224, 303)	274	(240, 318)
MVPA time (min/day)	57	(33, 82)	67	(48, 93)	105	(76, 147)	71	(51, 95)	79	(60, 104)
MVPA time of bouts ≥10 min (min/day)	7.1	(1.0, 22.2)	11.3	3.4, 26.4)	45	(36, 70)	12	(4, 27)	13.7	(5.4, 32.9)
Vigorous physical activity (min/day)	0.5	(0.1, 1.1)	0.7	(0.3, 1.3)	0.9	(0.5, 1.5)	0.7	(0.3, 1.3)	0.8	(0.4, 1.4)
Inactive time (min/day)	721	(628, 812)	699	(617, 770)	625	(575, 721)	688	(613, 766)	655	(602, 739)
Sleep time (min/day)	414	(346, 468)	421	(346, 469)	436	(359, 470)	415	(348, 465)	421	(349, 465)
Mean acceleration per 24 h (mg)	19.8	(15.8, 25.7)	22	(18, 27)	27	(22, 32)	23	(19, 27)	24	(21, 28)
Minimal acceleration during 2 most active minutes (mg)	273	(211, 333)	295	(244, 351)	311	(271, 377)	300	(246, 354)	314	(257, 368)
Minimal acceleration during 30 most active minutes (mg)	127	(103, 152)	136	(117, 160)	171	(150, 186)	139	(122, 163)	145	(130, 170)
Minimal acceleration during 60 most active minutes (mg)	97	(77, 120)	106	(90, 126)	137	(122, 153)	107	(92, 127)	114	(101, 131)
Daily steps (steps/day)	10,463	(7391, 13837)	11,966	(9,594, 14,925)	15,036	(11,702, 19,008)	12,186	(9,986, 15,047)	13,427	(11,833, 15,875)
Mean cadence per 24 h (steps/min)	7.2	(5.1, 9.5)	8.2	(6.6, 10.3)	10.5	(8,1, 13.1)	8.4	(6.9, 10.4)	9.3	(8.1, 11)
Cadence ≥100 steps/min (min/day)	8.7	(2.4, 18.3)	11.5	(4.2, 20.9)	32	(19, 49.6)	12.1	(4.5, 21.5)	13.8	(5.8, 28)
Time with cadence = 0 (%)	69	(64, 75)	68	(63, 72)	65	(62, 71)	67	(62, 71)	64	(61, 68)
Cadence of most active minute (steps/min)	106	(100, 112)	108	(103, 113)	113	(109, 117)	109	(104, 114)	110	(106, 114)
Cadence of most active 30 min (steps/min)	83	(72, 94)	88	(79, 96)	101	(95, 106)	89	(80, 97)	92	(84, 100)
Cadence of most active 60 min (steps/min)	72	(60, 83)	77	(67, 86)	93	(85, 99)	78	(69, 86)	82	(73, 90)

Parameters are indicated as group medians and first and third quartiles (in brackets) based on patients’ means over the measuring period. Inactive time: minutes with accelerations <25 mg; MVPA (moderate to vigorous physical activity): minutes with acceleration ≥100 mg.

### Daily activity measurements

When expressed as mean daily activity over the 18 days, 226 participants (80.1%) had ≥30 min of MVPA on an average day (Table 1). However, only 52 (18.4%) study participants spent at least 30 min in MVPA with bouts of ≥10 min, thus fulfilling 2010 WHO PA guideline recommendations. The median duration of all participants’ mean MVPA time was 57 (33, 82) minutes, and the median of each participant’s mean time in bouts ≥10-min MVPA was 7 (1, 22) minutes. Median sleep time was 6.9 (5.8, 7.8) hours, and median inactive time was 12.0 (10.5, 13.5) hours. One-hundred-and-fifty-five participants (55.0%) reached ≥10,000 steps/day, and 209 (74.1%) performed ≥7,500 steps/day. Two-hundred-and-four participants (72.3%) reached a cadence of ≥100 steps/min during the most active minute of the average day. Over the most active 30 min, this cadence was reached by 38 participants (13.5%), and over the most active 60 min, 12 participants (4.3%) reached this threshold.

**FIGURE 2:**
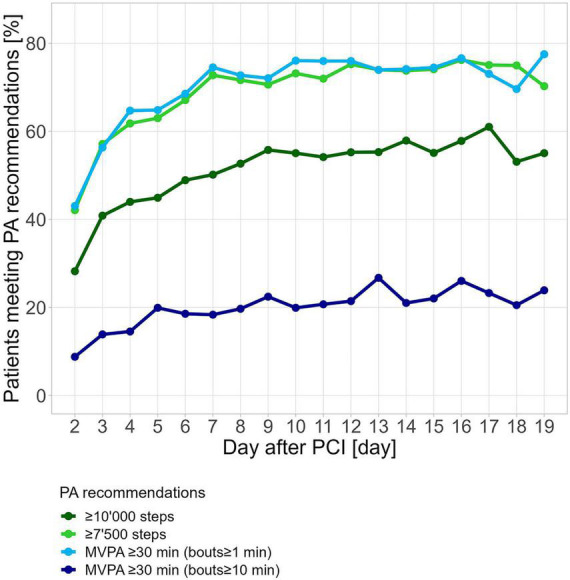
Percentage of participants reaching various criteria for PA after hospital discharge for PCI. PCI was performed on day 0. Fulfillment of PA criteria was calculated for each participant and each day individually.

On day 2 after PCI, 43.5% of participants with available data on that day fulfilled the MVPA criterion of at least 30 min according to 2020 WHO PA guidelines. This percentage increased steadily until day 7, after which it decreased again slightly (Figure 2). A similar percentage of participants fulfilled the criterion of a minimum of 7,500 steps/day. The minimum recommendation of 30 min of MVPA in ≥10-min bouts according to the 2010 WHO guideline was fulfilled by 8.9% on the second day and increased steadily until day 17 when 23.4% fulfilled this criterion. On day two, 28.5% reached 10,000 steps/day and by day 17, 61.3% had reached 10,000 steps/day.

### Linear regressions for moderate- to vigorous-intensity physical activity and daily steps

**FIGURE 3:**
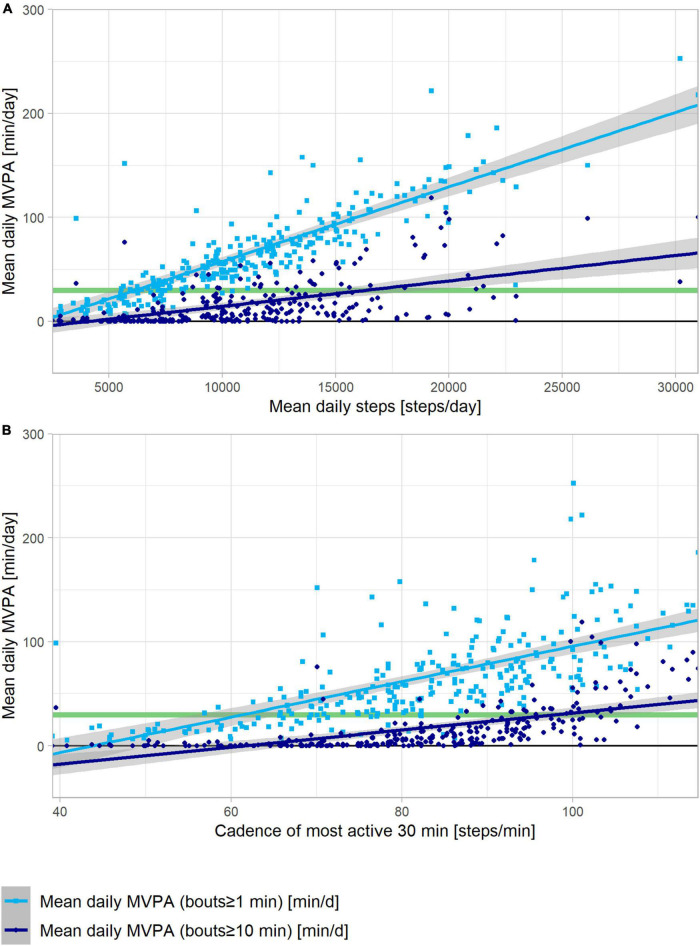
Linear regressions of MVPA in bouts of ≥1 min (light blue squares) and ≥10 min (dark blue dots) versus steps per day **(A)** and versus step cadence of the 30 most active minutes of each day **(B)**. Each data point reflects mean values over the 18 days for each patient. The 95% confidence interval is depicted by the gray area around the regression lines.

The linear regression of daily mean steps with daily mean MVPA according to the 2020 PA guidelines explained 47.5% of the total variability (*r* = 0.69, *p* < 0.0001), while the linear regression of daily mean steps with daily mean MVPA according to the 2010 PA guidelines explained only 13.6% (*r* = 0.37, *p* < 0.0001, Figure 3A). Approximately 2,500 steps corresponded to 0-min MVPA per 2020 WHO guidelines, and 5,000 steps corresponded to 0-min MVPA per 2010 WHO guidelines. The intersection of the regression line with 30 min of daily MVPA according to 2020 WHO guidelines corresponded to 6,250 daily steps, while more than 15,000 steps on average were necessary to reach the 30-min threshold according to 2010 WHO guidelines. Similar observations could be made for the linear regression models of the daily mean cadence of the most active 30 min with daily mean MVPA according to the 2010 and 2020 WHO guidelines (Figure 3B). The linear regression models for mean cadence with MVPA according to the 2010 guidelines explained 29.1% of the total variance (*r* = 0.54, *p* < 0.0001) and only 16.8% (*r* = 0.41, *p* < 0.0001) with MVPA according to the 2020 guidelines. The intersection of the regression line with 30 min of daily MVPA per 2020 WHO guidelines corresponded to 60 steps/min, while a cadence of 100 steps/min was observed for reaching the 30-min threshold according to 2010 WHO guidelines.

### Adherence to guidelines and steps per day according to age, sex, and coronary artery disease presentation

Overall, we found that most of the proportions of adherence did not statistically differ across categories of age, sex, and disease presentation at the PCI (Table 2). However, the lowest adherence to the 2010 WHO guidelines was observed among women (8.9%), and patients older than 62 years had a lower proportion (74.5%) of adherence to the 2020 guidelines, compared to the patients in the younger group (86%).

**TABLE 2:** Results of adherence to guidelines and steps per day according to age, sex, and disease presentation.

	Total sample	Age groups		Sex		Coronary disease presentation	
		<62	≥62	Male	Female	ACS	CCS
Number of patients [*n* (%)]	282	141 (50)	141 (50)	237 (84)	45 (16)	249 (88)	33 (12)
**Adherence to guidelines and daily steps [*n* (%)]**							
WHO 2020 guidelines	226 (80.1)	121 (86)	105 (74.5)*	192 (81)	34 (75.6)	202 (81)	24 (73)
WHO 2010 guidelines	52 (18.4)	31 (22)	21 (15)	48 (20.3)	4 (8.9)*	44 (18)	8 (24.2)
Steps ≥7,500	210 (74.5)	110 (78)	100 (71)	177 (74.7)	33 (73.3)	186 (74.7)	24 (73)
Steps ≥10,000	154 (54.6)	77 (54.6)	77 (54.6)	127 (53.6)	27 (60)	136 (54.6)	18 (54.5)

*n*, number of participants; ACS, acute coronary syndrome; CCS, chronic coronary syndrome; WHO, World Health Organization. Percentages are based on the population for each column.

*p-value < 0.05 for the comparison between groups of age, sex, and coronary disease presentation.

## Discussion

**FIGURE 4:**
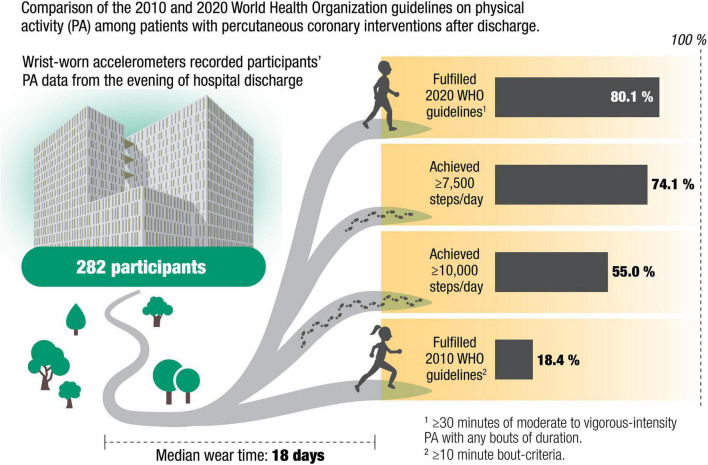
Illustration of fraction of patients reaching 2020 versus 2010 World Health Organization guidelines on physical activity, as well as those reaching 7,500 and 10,000 steps per day.

After recent PCI, PA assessment with wrist-worn accelerometers among our participants was found to be highly feasible with a participation rate of 40 and 87% compliance. We found a wide variation in the prevalence of sufficient activity according to WHO PA guidelines from 2010 and 2020, namely spending 30 min in MVPA with or without 10-min bouts. While only 18% of our participants fulfilled 2010 WHO guidelines with MVPA counted only as bouts lasting at least 10 min, 80% met the recommendations from the 2020 WHO guidelines. A higher median number of daily steps and more daily min at a cadence ≥100 steps/min was found among participants who reached the average of 30-min MVPA in 10-min bouts when compared to participants who only met the recommendations from the 2020 WHO guidelines (Figure 4).

To our knowledge, this study is the first to quantify the discrepancy between the achievement of PA recommendations with and without the 10-min bout requirement in patients after PCI. Our findings are consistent with other studies conducted on different populations. For instance, a cross-sectional study investigating data from the 2003–2004 National Health and Nutrition Examination Survey (NHANES) (37) and a study on data from the Framingham Heart Study (38) also reported a fourfold discrepancy, whereas a study on a subsample of the NHANES population found a sixfold discrepancy (39). The 2020 WHO guideline was based on studies claiming that bouts shorter than 10 min of MVPA were also associated with reduced all-cause mortality in the general population (15). However, the majority of those studies supporting the health benefits of PA accumulated in bouts of <10 min in duration used a cross-sectional design, with none of the randomized studies reporting on the effects of PA accumulated in bouts of <10 min. Other studies established associations of MVPA acquired sporadically or in bouts ≥10 min with some cardiovascular risk factors. For instance, a study of >1,000 Canadian adults wearing hip-worn tri-axial accelerometers, reported that the time of MVPA with bouts ≥1 min was nearly double the time of MVPA with bouts ≥10 min (40). The presence or absence of metabolic syndrome was equally well discriminated by bouted (≥10-min) or sporadic (1–9 min) MVPA (40). Similar associations of CVD factors with MVPA bouts duration were found in a sample of >2,000 participants from the Framingham Heart Study (38). In another study of over 6,000 adults from the NHANES study, MVPA in bouts and non-bouts were similarly associated with cardiovascular risk factors (37); however, a study among the subpopulation of adults younger than 65 years from the Canadian health measures survey found a four times greater inverse association of obesity with MVPA in bouted compared to sporadic MVPA (39). The Coronary Artery Risk Development in Young Adults (CARDIA) study of approximately 2,000 healthy adults found that accumulating sporadic MVPA, independently of bouts, was a protective factor against the development of hypertension but not against obesity (41).

Bouts of 10 min or longer are likely to represent planned and structured exercise, while shorter bouts more likely reflect activities of daily living. Likewise, the median time with a cadence ≥100 steps/min of our study participants was 8 min/day, indicating many of our participants barely reached this cadence. Hence, most of our participants’ steps were performed at low cadences or in bouts shorter than 1 min, which again suggests activities of daily living rather than physical exercise increasing heart rates and cardiac output. In our study, the proportion of participants fulfilling the 2020 WHO PA guidelines was slightly higher than the proportion of participants walking ≥7,500 steps/day—a threshold found to discriminate between cardiovascular risk factors (6). The percentage of participants walking ≥10,000 steps was between the proportions of participants fulfilling the 2010 and 2020 WHO PA guidelines. Unlike MVPA, people can easily verify the number of steps calculated by an accelerometer device by walking a predefined number of steps or by walking at a certain cadence for a defined time. Not only can the number of steps be verified, but it is also an easily followed recommendation, such as walking 3,000 steps or walking at brisk 100 steps/min for 30 min.

It is questionable whether PA of very short duration has the same beneficial effects on patients with CVD as structured exercise. Several mechanisms may explain the known benefits associated with PA in patients with CVD, including endothelial function improvement (42, 43) and antiatherosclerotic (43, 44) and anti-inflammatory (45) effects. Traditional risk factors for CHD such as diabetes, hypertension, smoking, and hypercholesterolemia are associated with endothelial dysfunction, which in turn results in impaired nitric oxide production, abnormal vasoconstriction, chronic inflammation, and increased oxidative stress (46). Endothelial dysfunction, inflammation (47), and oxidative stress (48) play an important role in both the pathogenesis and prognosis of CVD. Against this background, PA increases beneficial shear stress at the vessel wall, down-regulates the expression of the angiotensin II type 1 receptor (49), and decreases NADPH oxidase activity and superoxide anion production, which in turn decreases the generation of reactive oxygen species and inflammation while preserving endothelial nitric oxide bioavailability and its protective anti-atherosclerotic effects (50). Conversely, physical inactivity increases vascular NADPH oxidase activity and increases vascular reactive oxygen species generation, which in turn contributes to endothelial dysfunction and atherosclerosis (51). Exercise training of distinguished volume and intensity has proven beneficial effects on endothelial function and arterial stiffness (52, 53). At least for weight loss and prevention of obesity, bouts ≥10 min have been suggested as necessary (39, 41, 54). Future studies need to clarify how recommendations are actionable to patient benefit and whether daily step targets for patients after PCI gauge prognostic importance.

### Limitations

Some limitations may affect our study. First, inactive and uninterested patients may have been lost during recruitment since participants’ consent required their willingness to wear an accelerometer. Consequently, our study participants may be more active and compliant than typical patients after PCI in clinical settings. With a 40% inclusion rate, it is possible that our study included a higher percentage of physically active patients whereas inactive patients could have refused participation. However, after the recommendation for monitoring objective PA that has been recently endorsed by the ESC, (55) the inclusion process for this and any other future studies is expected to improve. Specifically in our setting, the use of accelerometer is now a standard of care. All patients are recommended to wear the accelerometer for 18 days after hospital discharge from PCI and together with their general practitioners receive their analyzed data and PA recommendations upon returning the device.

Our recruitment team did not enlist patients who did not qualify for ambulatory cardiac rehabilitation because they were too frail or cognitively impaired. Therefore, our results may have been affected by selection bias. However, selection bias did not affect the large discrepancy between the number of participants satisfying 2010 versus 2020 WHO PA guideline criteria, which was our main aim. Second, the median MVPA of 1-min bouts among our study participants was 57 min/day or 399 min/week, fulfilling or even exceeding the recommended range of 150–300 min/week. It is possible that PA measured in our study overestimated PA levels due to the Hawthorne effect since pedometer use has been shown to increase patients’ PA (19, 22). Wrist-worn accelerometers might also underestimate activities, such as cycling (56). In contrast, activities involving arm movements may overestimate PA levels since the metabolic cost of arm movements is smaller than that of leg movements due to the smaller muscle mass involved in the effort (57). However, since walking is one of the most frequently reported leisure time activities worldwide, this limitation may be negligible (58), especially among patients with cardiac conditions (6).

Third, since most PA data are averaged over 1-min windows, dropping the criterion of 10-min bouts means that bouts as few as 1 min are sufficient for qualifying as MVPA in the 2020 WHO guidelines. However, with many proprietary devices, the minimal bout length is not obvious to the user, and some devices use 15 s or even 5 s epochs (59). The choice of epoch length also affects the calculated daily time spent with MVPA. MVPA time was doubled when epoch length was increased from 4 to 20 or 60 s in a study using hip-worn uni-axial accelerometers (60). Unless a device with a defined wearing location, data sampling rate, epoch duration, and algorithm settings for calculation of MVPA is validated against energy consumption measured by a metabolic cart, it is impossible to know whether time with MVPA is actually time with an energy consumption ≥3 METs.

Our data imply that tracking the global target set by WHO to reduce inactivity by 2025, should take into consideration the discrepancy of values that are consistently reported in the literature. Using the new guidelines to evaluate policies supporting PA in settings where baseline PA levels were measured through different criteria, may be biased and not reflect the reality of the expected change. Finally, whether CV risk can be equally reduced by MVPA with and without the 10-min bout requirement in patients after PCI needs to be investigated in future studies, such as the PIPAP study. Since the identification of MVPA is highly dependent on the duration of analyzed bouts and consequently varies between accelerometer devices and algorithm settings, a target number of steps may be more manageable, understandable, and feasible for people.

## Conclusion

This study found a fourfold discrepancy in the frequency of participants fulfilling 2010 and 2020 WHO guidelines for PA among patients following hospital discharge after PCI. In this setting, the recommendations from the 2020 WHO PA guidelines for MVPA were fulfilled easily by activities of daily living, without any planned or structured exercise. Future studies need to clarify how recommendations are actionable to patient benefit and whether daily step targets for patients after PCI gauge prognostic importance.

## Data availability statement

The raw data supporting the conclusions of this article will be made available by the authors, without undue reservation.

## Ethics statement

The studies involving human participants were reviewed and approved by the Ethikkommission des Kantons Bern. The patients/participants provided their written informed consent to participate in this study.

## Author contributions

NG-J, AB, OF, and MW designed the study. PE, SW, RF, and JF were involved in data collection, processed the data, and performed data analyses. PE, SW, and NG-J drafted the manuscript. All authors approved the final version of the manuscript.
