# Erratum: Objectively measured adherence to physical activity among patients with coronary artery disease: Comparison of the 2010 and 2020 World Health Organization guidelines and daily steps

**DOI:** 10.3389/fcvm.2023.1173511

**Published:** 2023-03-08

**Authors:** Frontiers Production Office

**Affiliations:** Frontiers Media SA, Lausanne, Switzerland

**Keywords:** physical activity, guidelines, accelerometer, percutaneous coronary intervention, step counting

An Erratum on Objectively measured adherence to physical activity among patients with coronary artery disease: Comparison of the 2010 and 2020 World Health Organization guidelines and daily steps By Eser P, Gonzalez-Jaramillo N, Weber S, Fritsche J, Femiano R, Werner C, Casanova F, Bano A, Franco OH and Wilhelm M. (2022) Front. Cardiovasc. Med. 9:951042. doi: 10.3389/fcvm.2022.951042

An omission to the funding section of the original article was made in error. The following sentence has been added: “Open access funding was provided by the University of Bern.”

The original version of this article has been updated.

